# Spatio-temporal spread of Lassa virus and a new rodent host in the Mano River Union area, West Africa

**DOI:** 10.1080/22221751.2023.2290834

**Published:** 2023-12-04

**Authors:** Umaru Bangura, Christopher Davis, Joyce Lamin, James Bangura, Barré Soropogui, Andrew J. Davison, Jenna Nichols, Matej Vucak, Mickael Dawson, Rashid Ansumana, Dianah Sondufu, Dániel Cadar, Toni Rieger, Emma Thomson, Foday Sahr, N’Faly Magassouba, Bruno Ghersi, Brian H. Bird, Elisabeth Fichet-Calvet

**Affiliations:** a Bernhard Nocht Institute for Tropical Medicine, WHO Collaborating Centre for Arbovirus and Hemorrhagic Fever Reference and Research, Hamburg, Germany; b MRC-University of Glasgow Centre for Virus Research, Glasgow, UK; c Mercy Hospital Research Laboratory, Bo, Sierra Leone; d University of Makeni and University of California, Davis One Health Program, Makeni, Sierra Leone; e Laboratoire des Fièvres Hémorragiques en Guinée, Conakry, Guinea; f College of Medicine and Allied Health Sciences, University of Sierra Leone, Freetown, Sierra Leone; g One Health Institute, School of Veterinary Medicine, University of California, Davis, Davis, CA, USA

**Keywords:** Lassa virus, spread, phylogeography, rodent, *Lophuromys sikapusi*, Guinea, Sierra Leone, West Africa

## Abstract

The spread of Lassa virus (LASV) in Guinea, Liberia and Sierra Leone, which together are named the Mano River Union (MRU) area, was examined phylogeographically. To provide a reliable evolutionary scenario, new rodent-derived, whole LASV sequences were included. These were generated by metatranscriptomic next-generation sequencing from rodents sampled between 2003 and 2020 in 21 localities of Guinea and Sierra Leone. An analysis was performed using BEAST to perform continuous phylogeographic inference and EvoLaps v36 to visualize spatio-temporal spread. LASV was identified as expected in its primary host reservoir, the Natal multimammate mouse (*Mastomys natalensis*), and also in two Guinean multimammate mice (*Mastomys erythroleucus*) in northern Sierra Leone and two rusty-bellied brush-furred mice (*Lophuromys sikapusi*) in southern Sierra Leone. This finding is consistent with the latter two species being secondary host reservoirs. The strains in these three species were very closely related in LASV lineage IV. Phylogenetic analysis indicated that the most recent common ancestor of lineage IV existed 316–374 years ago and revealed distinct, well-supported clades from Sierra Leone (Bo, Kabala and Kenema), Guinea (Faranah, Kissidougou-Guekedou and Macenta) and Liberia (Phebe-Ganta). The phylogeographic scenario suggests southern Guinea as the point of origin of LASV in the MRU area, with subsequent spread to towards Mali, Liberia and Sierra Leone at a mean speed of 1.6 to 1.1 km/year.

## Introduction

Over the last century, increasing human pressure on land resources and the amount and pace of migration flows have linked pathogens with new hosts. Together with expanding human populations, this has contributed to an increase in emerging and re-emerging epidemics [1,2]. Environmental factors such as temperature, precipitation and the existence of pastures are thought to influence ecological suitability for virus circulation [3,4]. Our understanding of the evolution and dynamics of emerging zoonotic viruses continues to expand due to advances in molecular sequencing technologies and phylogenetic approaches. In the last decade, phylogeographic inference has gained popularity as a tool for analysing the evolutionary dynamics and timing of virus epidemic spread [5-7].

One prominent zoonotic virus, Lassa virus (LASV; *Mammarenavirus lassaense*) is an arenavirus that causes a viral haemorrhagic illness in humans known as Lassa fever (LF). This disease has been recognized since the 1950s, and the virus was first identified in 1969 in the village of Lassa, Nigeria [8]. Since its discovery, regular LF outbreaks have occurred in Benin, Guinea, Liberia, Sierra Leone, and Nigeria [9-12]. An estimated 897,700 people are yearly infected by LASV, with 18,000 deaths occurring in the West Africa region [13].

LASV shows a high degree of genetic diversity, with seven distinct phylogenetic lineages known to be circulating in West Africa. Strains in Nigeria comprise lineages I, II, III and VI and are more diverse than those in the Mano River Union (MRU) area, which includes countries bordering the Mano River such as Guinea, Liberia and Sierra Leone, where they are categorized as lineage IV. Strains from Côte d'Ivoire and Mali are designated as lineage V [14-16], and the Benin/Togo strain, which was isolated from a nosocomial outbreak in 2014–2016, is classified as lineage VII [17].

The Natal multimammate mouse (*Mastomys natalensis*) was previously thought to be the sole host reservoir for LASV [9,18]. However, recent discoveries have also implicated the Guinean multimammate mouse (*Mastomys erythroleucus*) in Guinea and Nigeria, the African wood mouse (*Hylomyscus pamfi*) in Nigeria [19], and the pygmy mouse (*Mus baoulei*) in Benin and Ghana [20]. In the context of a growing interest in ecological studies and the development of powerful diagnostic tools over the last 20 years, these findings indicate that several species may be involved in LASV transmission.

According to previous phylogenetic studies, LASV originated in Nigeria a thousand years ago and spread westward across West Africa. LASV first appeared in the MRU area 300–350 years ago, with the likely entry point in Liberia [21-23]. In Sierra Leone, many discrete LF outbreaks may have occurred in endemic areas prior to the discovery of the virus. For instance, a potential LF outbreak may have occurred in 1956 at the Nixon Memorial Hospital in Segbwema, in the eastern region [24]. Since the 1970s, there have been multiple LF outbreaks, with hundreds of cases generally being reported in the eastern region [12,25]. In Guinea, very few acute human cases of LF have so far been reported, most occurring in southern Guinea [26,27].

Ecological studies in Sierra Leone and Guinea have revealed the circulation of LASV in *M. natalensis*, primarily in the south and northern regions of Sierra Leone and also in Guinea along the border with Sierra Leone [22,28-29]. Despite these studies, LASV evolution in the two countries is still unclear as, due to the lack of rodent-derived LASV sequences, the great majority of analyses have focused on human-derived LASV sequences. Here, we aimed to provide a detailed scenario focusing on rodent-derived sequences. As *M. natalensis* live in a small home range of around 300–600 m^2^ [30], they provide a high degree of geographical accuracy. This is not the case with humans, who sometimes travel long distances. For example, the origin of the “AV” strain described in a German tourist who had travelled between Côte d'Ivoire and Burkina Faso remained unclear for 10 years. Only the discovery of LASV-positive *M. natalensis* in southern Mali and then northern Côte d'Ivoire has enabled these sequences to be compared with those observed in the German tourist, suggesting that she was infected in this region rather than in Burkina Faso [31]. Similarly, the acute case of a forester recently discovered in eastern Côte d'Ivoire was probably acquired far from home, as he was infected by a strain that is phylogenetically close to those observed in Upper Guinea [32]. The aim of our study was to investigate the LASV spread and circulation in the MRU area by using rodent-derived, whole LASV genome sequences from 27 localities. By adding these sequences to existing human-derived LASV sequences, the prospect was of identifying a well-supported route for LASV propagation in Guinea and Sierra Leone.

## Materials and methods

### Origin of rodent-derived LASV sequences

The LASV sequences generated in this study originated from three research programmes entitled: (i) “New approaches to the treatment and control of Lassa fever and yellow fever in West Africa (TREATCONTROL-VHF)” funded by the European Commission; (ii) “Lassa fever in Guinea and Sierra Leone: rodent control and seasonality of human exposure to rodents (LAROCS)” funded by the Deutsche Forschungsgemainschaft; and (iii) “Preventing Emerging Pathogenic Threats (PREEMPT)” funded by the Defense Advanced Research Projects Agency, USA.

**Figure 1.**
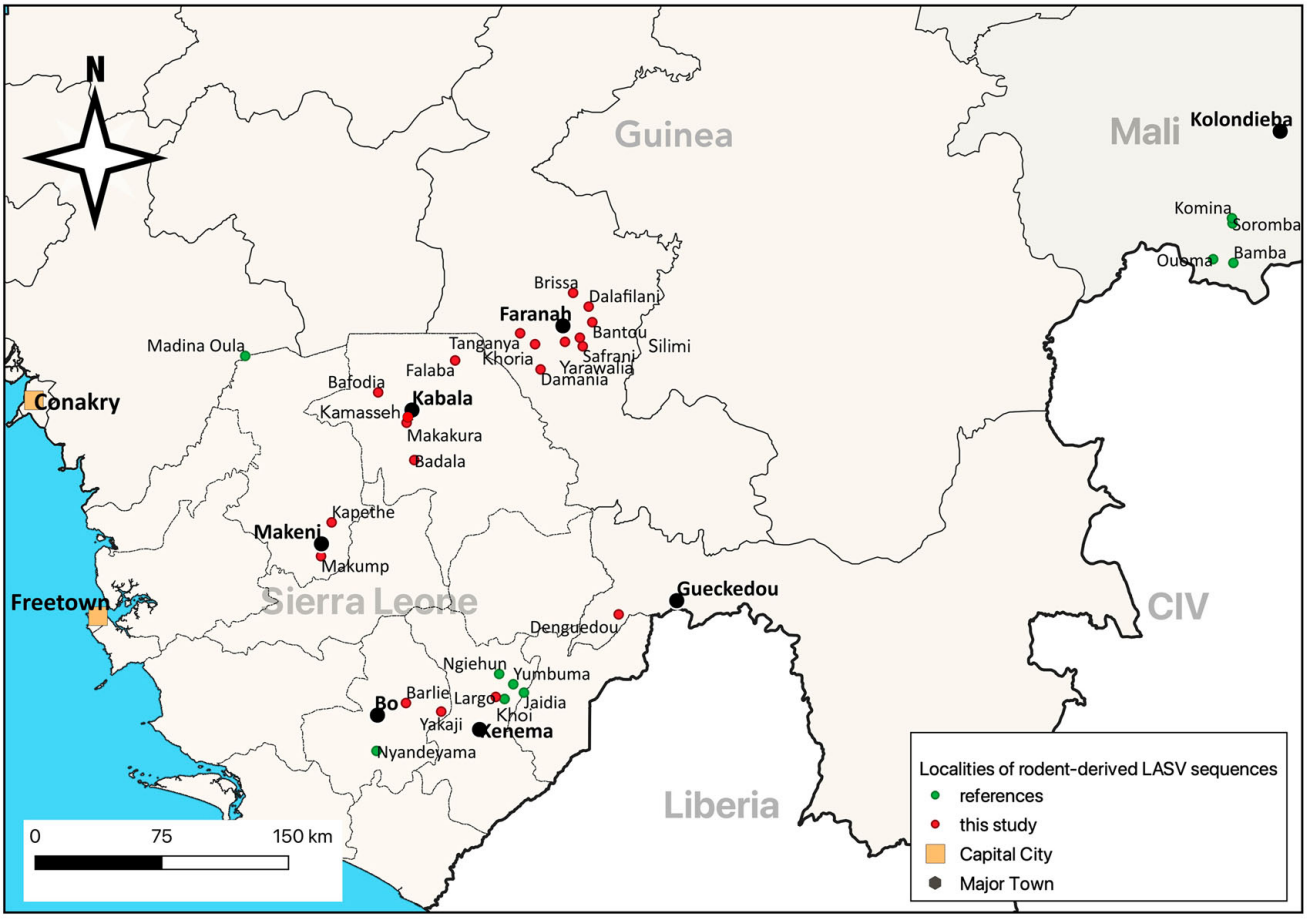
Map showing the localities where LASV rodent-derived sequences were generated in Guinea, Mali and Sierra Leone. Red dots: sequences provided in this study, green dots: sequences already published (Tables S2–S4).

The rodents were sampled between 2003 and 2020 in Guinea and Sierra Leone in 21 localities. In Guinea (TREATCONTROL-VHF and LAROCS programmes), they came from 9 localities located around Faranah in Upper Guinea and one locality in Gueckedou in Forest Guinea. This represented a trapping effort of 5,676 trap nights. The villages had populations of 600–1500 and were located in rural areas within a 45-minute drive of each other. The rodents were captured using Sherman live traps (H.B. Sherman Trap Co., Tallahassee, FL, USA) set in houses and surrounding habitats for 3 days (for a detailed explanation concerning the trapping methodology, see Fichet-Calvet et al. 2007, 2014). In Sierra Leone (LAROCS and PREEMPT programmes), the rodents come from 11 localities around Bo, Kabala, Kenema and Makeni. In the LAROCS programme, rodents were sampled in the same way as in Guinea and represented a trapping effort of 2842 trap nights. In the PREEMPT programme, rodents were sampled inside and outside dwellings, representing a trapping effort of 4278 trap nights. Figure 1 shows the localities with rodent-derived sequences.

### LASV testing and identification of host species

TREATCONTROL-VHF: RNA was extracted from whole blood samples using a QIAamp Viral RNA Mini kit (Qiagen). LASV RNA-positive samples were identified by conventional RT–PCR targeting a region of the RNA polymerase gene in the L segment (Table S1, [33]). DNA was extracted from liver samples using a DNeasy Blood & Tissue kit (Qiagen). The host species of LASV-positive rodents were identified in using a specific PCR targeting the mitochondrial gene encoding cytochrome b [34].

LAROCS: RNA was extracted from a range of samples (dried blood spot, whole blood) using a QIAamp Viral RNA Mini kit (Qiagen). LASV RNA-positive samples were identified by conventional RT–PCR using a One-Step RT–PCR kit (Qiagen) targeting regions of the glycoprotein (GP) gene in the S segment and the RNA polymerase gene in the L segment [34]. Initial testing was performed using samples pooled in threes. Final testing was performed using individual samples in LASV-positive pools. The host species of LASV-positive rodents were identified as described above.

PREEMPT: Nucleic acids were extracted from a range of samples (oral swab, urogenital swab, blood, liver, lung and spleen), as described previously [35]. LASV RNA-positive samples were identified by quantitative RT–PCR targeting regions of the GP gene in the S segment and the L gene in the L segment, using a RealStar Lassa Virus RT–PCR kit 2.0 (Altona Diagnostics). The host species of LASV-positive rodents were identified by sequencing the mitochondrial gene encoding cytochrome b from the read datasets used to sequence LASV genomes.

### Sequencing of LASV strains

LASV genomes in LASV-positive samples were determined in each research programme using metatranscriptomic next-generation sequencing (NGS) protocols.

TREATCONTROL-VHF: Two LASV strains were cultivated in Vero cells in a BSL-4 laboratory at the Bernhard Nocht Institute for Tropical Medicine (BNITM), Hamburg, Germany. The strains were sequenced from RNA extracts obtained from infected cell culture supernatants, as described previously [20]. Briefly, supernatants were filtered and digested with a mixture of nucleases. RNA was extracted from viral particles and processed into sequencing libraries, which were sequenced on the NextSeq 550 platform (Illumina). LASV sequences were refined iteratively by aligning reads to a reference sequence to create an improved reference sequence.

LAROCS: RNA extracts were obtained using linear acrylamide (Invitrogen) to replace the carrier RNA and sent to the MRC-University of Glasgow Centre for Virus Research, for metagenomic sequencing with the Illumina MiSeq platform as described previously [22]. With the de novo assembly, Fastq files from the Illumina procedure were reference mapped across full-genome LASV using Tanoti, (http://www.bioinformatics.cvr.ac.uk/tanoti.php). In addition to metagenomic sequencing, we used Sanger sequencing after the initial screening (PCR products from the GP and polymerase diagnostics) and completed some gaps with specific primers when necessary (Table S1). Reads generated with both Sanger and NGS were then assembled for each LASV variant using MacVector v18.5.1 software (2022 Mac Vector, Inc., Apex, NC, USA).

PREEMPT: LASV sequences were determined as described previously [35]. Briefly, RNA was purified, depleted of rRNA and processed into sequencing libraries, which were analyzed on the NextSeq 550 platform (Illumina). LASV sequences were determined by assembling the reads into contigs and joining contigs into genome sequences manually by iteratively incorporating additional reads sharing short sequences near the contig ends.

### Sequence alignment

The consensus LASV sequences from the MRU area obtained by others previously and in this study were aligned using MacVector v18.5.1 (2022 Mac Vector, Inc., Apex, NC, USA).

### Phylogeographic analysis

To analyze the protein-coding regions of the GP and NP genes from the same S segment (in which these genes are located in opposite orientations), each NP sequence was reverse complemented and attached at the 3’-end of the corresponding GP sequence with three unspecified nucleotides (NNN) between the two sequences to keep them in frame. It was therefore possible to assign the position of each nucleotide (1, 2 or 3) in each codon to get a better estimation of the mutation rate. The protein-coding region of the polymerase gene was analyzed separately.

To understand the evolutionary dynamics and spread pattern of LASV in the MRU area, continuous phylogeographic inference was performed using BEAST v1.10.4 [36]. The “general time-reversible” (GTR) model of sequence evolution with a gamma distribution of among-site nucleotide substitution rate variation (GTR + gamma) was identified as the substitution model that best described the datasets. A Cauchy relaxed random walk (RRW) diffusion model was adopted to generate a posterior distribution of phylogenetic trees having interior nodes. Each sequence was tagged with geographical coordinates (latitude and longitude). The analyses employed a constant size coalescent model as the tree topology prior, and an uncorrelated relaxed molecular clock to examine the evolutionary relationships among the sequences [37]. The Markov chain Monte Carlo (MCMC) chains were run for 500 million iterations and sampled every 50,000 states to obtain an effective sample size (ESS) of >200 for all the parameters for each dataset; segment S (GP + NP) and segment L (polymerase). The quality of the analysis was assessed using Tracer v1.7.1 [38] with log files containing 1001 states and 10% burn-in. A consensus tree was obtained using TreeAnnotator v1.10.4 [39] run with the maximum clade credibility tree option. The tree graphics were adjusted manually using FigTree v1.4.4 (http://tree.bio.ed.ac.uk/software/figtree/). EvoLaps v36 (https://www.evolaps.org, [40]) was used to visualise the spatio-temporal spread of LASV from phylogenetic trees associated with localities.

## Results

### LASV sequences generated

A total of 35 complete and 5 partial sequences of the S segment and 16 complete and 8 partial sequences of the L segment were generated. For the 40 S segment sequences, 13 were derived from rodents trapped in Guinea and 27 were derived from rodents trapped in Sierra Leone. For the 24 L segment sequences, 13 were derived from rodents trapped in Guinea and 11 were derived from rodents trapped in Sierra Leone (see Tables S2-S4 in Supplementary Information).

### New reservoir hosts for Lassa virus in Sierra Leone

**Figure 2.**
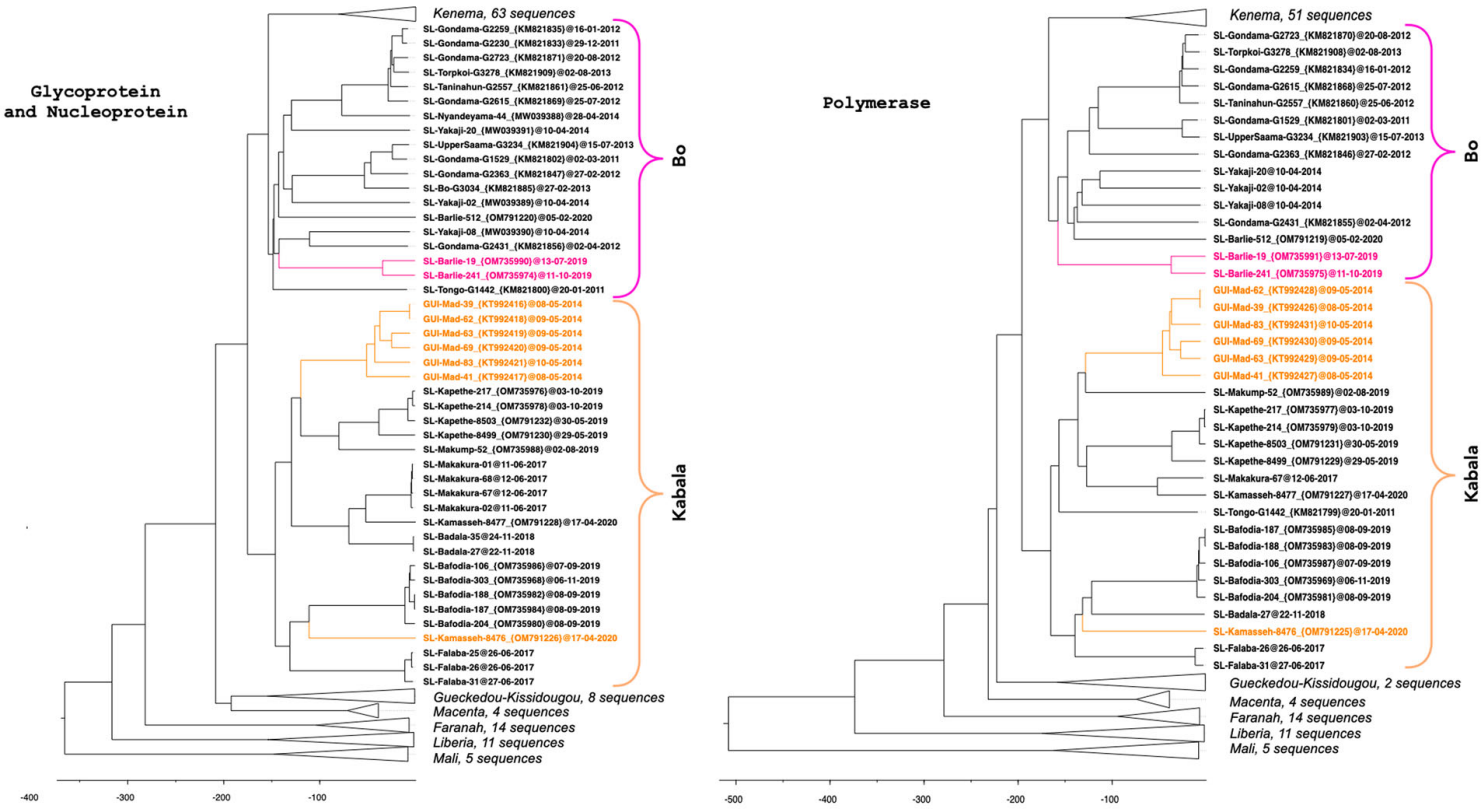
Time-calibrated phylogeny of LASV glycoprotein + nucleoprotein and polymerase sequences within lineages IV and V. Lineage V in Mali was used to root the tree. The analysis included 151 GP + NP sequences and 125 polymerase sequences. Sequences derived from new hosts are indicated in colour (*Lophuromys sikapusi* in pink and *Mastomys erythroleucus* in orange). Branches corresponding to clusters in Kenema in Sierra Leone, Kissidougou, Macenta, Faranah in Guinea, Liberia and Mali are collapsed (see Figures S1–S2 for details).

In June 2019 and October 2019, two *Lophuromys sikapusi* animals were sampled outside dwellings in Barlie in southern Sierra Leone, and found to be LASV-positive. In this locality, *L. sikapusi* (n = 13) was more abundant than *M. natalensis* (n = 9) when we consider the 4 trapping sessions from June 2019 to February 2020. Phylogenetic analyses of the GP + NP and polymerase genes revealed that these LASV strains, despite their presence in an unusual host, belong to the Bo clade with highly supported nodes (posterior probability = 1; Figure 2).

In April 2020, two *Mastomys* were sampled in Kamasseh, which is located near Makeni in central Sierra Leone, and found to be LASV-positive. One animal (*M. natalensis*) was trapped inside a dwelling and the other (*M. erythroleucus)* was trapped outside. In this locality, *M. natalensis* was more abundant (n = 8) than *M. erythroleucus* (n = 2). Phylogenetic analyses of the GP + NP and polymerase genes placed the *M. erythroleucus*-derived virus within a clade consisting of sequences from the Kabala area in northern Sierra Leone and Madina Oula in coastal Guinea, with highly supported nodes (posterior probability = 1; Figure 2). The *M. erythroleucus*-derived sequence clustered with *M. natalensis*-derived sequences from Kabala area with high supported nodes (posterior probability = 1, Figure 2). This finding represents the first detection of LASV in *M. erythroleucus* in Sierra Leone and validates the species’ ability to host the virus.

### Phylogeography of LASV

In order to investigate the evolutionary relationships among LASV strains in the MRU area, a time-calibrated phylogenetic reconstruction was performed for the S and L segments (as the GP + NP and polymerase sequences, respectively) using BEAST. LASV sequences from Sierra Leone, Guinea, and Liberia constitute lineage IV, while those from Mali constitute lineage V, which serves as the outgroup. The resulting estimate of the time of the most recent common ancestor (TMRCA) in lineage IV as 316–374 years ago. The analysis also showed three distinct and highly supported clades in Sierra Leone (Bo, Kabala and Kenema), three in Guinea (Faranah, Kissidougou-Guekedou and Macenta) and one in Liberia (Phebe-Ganta) (Figures S1-S2). The time-calibrated phylogeny of the S segment showed that the Bo and Kabala clades in Sierra Leone, the Phebe-Ganta in Liberia and the Kissidougou-Guekedou in Guinea emerged at approximately the same time, about 146–155 years ago (95% highest posterior density interval (HPD) = 125–186; Table 1). This is contemporary with the emergence of the Kolondieba clade in south Mali (TMRCA = 148 years; 95% HPD = 120–184). The most recent clades are located around Kenema in Sierra Leone and Macenta in Guinea (TMRCA = 71–80 years; 95% HPD = 65–91), and those located around Faranah emerged 104 years ago (95% HPD = 87–124). The time-calibrated phylogeny of the L segment showed similar but slightly greater TMRCA values than the S segment. Only the Faranah clade showed a smaller value.

**Table 1.** TMRCA values and 95% HPD intervals of the S and and L segments in LASV genomes according to country and locality. Values are in years.

Country	Locality	S segment (GP + NP)	L segment (polymerase)
Mali	Kolondieba	148 [54–118]	162 [67–135]
Liberia	Phebe-Ganta	154 [61–120]	174 [80–144]
Guinea	Faranah	104 [146–158]	94 [179–169]
	Macenta	71 [145–160]	74 [148–181]
	Gueckedou-Kissidougou	155 [63–121]	158 [66–123]
Sierra Leone	Kabala	146 [59–106]	165 [77–127]
	Bo	148 [63–103]	158 [72–115]
	Kenema	80 [152–150]	86 [158–155]

We noted that certain LASV strains detected in patients from Gondama in Sierra Leone were distributed in both the Bo (G1529, G2230, G2259, G2363, G2431, G2615 and G2723) and Kenema (806827, G1932, G2184 and G2903) clades. Similarly, variants from patients in Tongo were distributed in both clades. This may indicate active circulation of the virus between the two neighbouring provinces. However, there may also have been an error when entering data in the register, as both Bo and Kenema have villages named Gondama.

**Figure 3.**
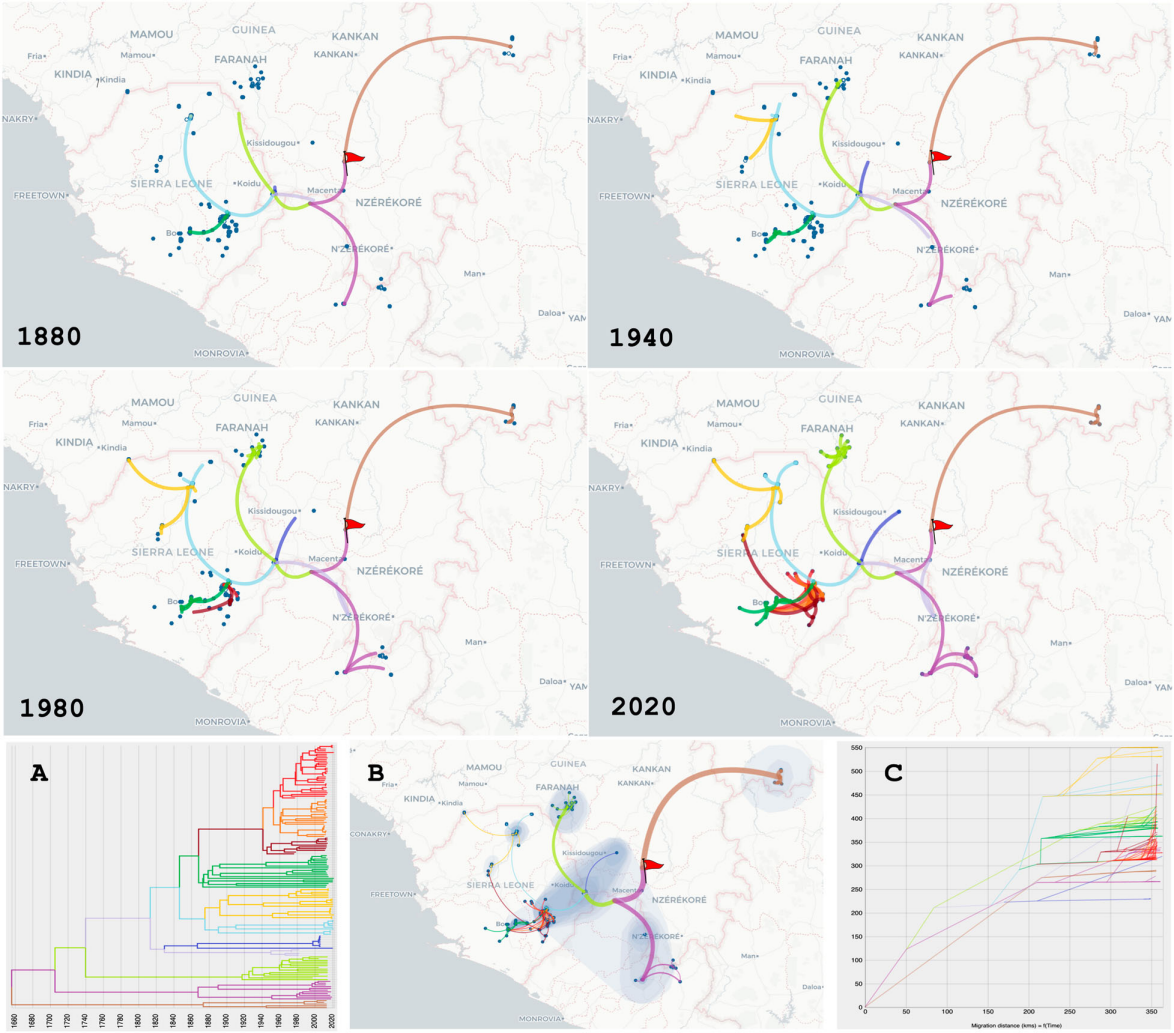
Phylogeographic scenario of LASV spread in Guinea, Liberia, Mali and Sierra Leone based on analysis of the S segment (GP and NP genes, 3186 nt). Upper four panels: Maps showing transition paths of LASV spread from 1880 to 2020, with the transition paths symbolised by curved lines coloured according to their cluster in A. The origin is marked by a red flag. A: Phylogenetic tree with branches in clusters coloured according to the transition paths. B: Map showing the global scenario with 80% of the highest posterior density (coloured blue). Transition paths are of varying thickness, with older paths represented by thick lines and newer paths by thin lines. C: Migration diagram showing distance in km on the *x* axis and time in years on the *y* axis. Migration routes are coloured according to the clusters in A.

**Figure 4.**
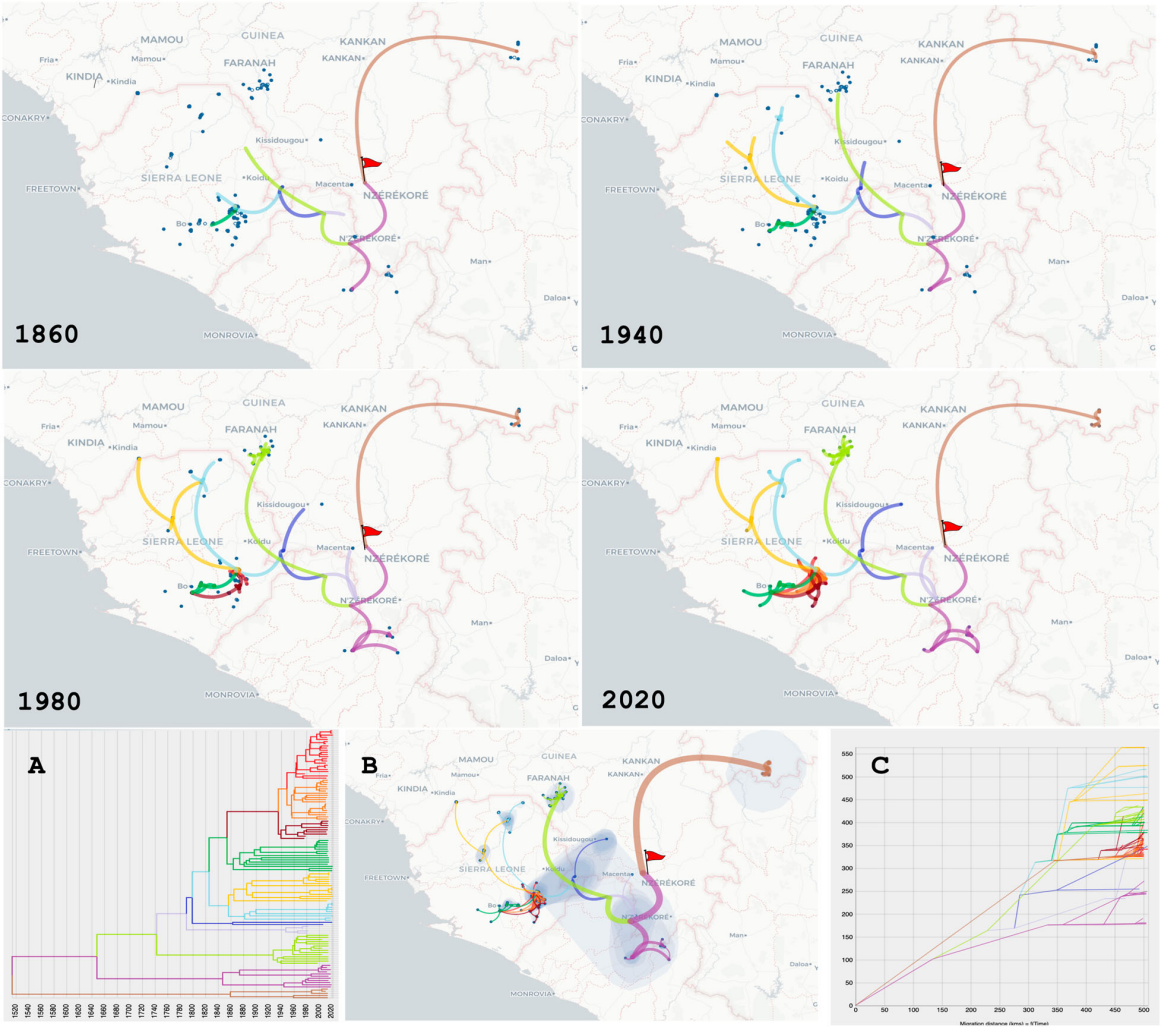
Phylogeographic scenario of LASV spread in Guinea, Liberia, Mali and Sierra Leone based on analysis of the polymerase (L segment, 6678 nt). See Figure 3 for details.

The phylogeographic scenario is represented on a map showing changes of locations using paths (Figures 3 and 4). According to our analysis, the point of origin would be located in southern Guinea, from where the virus would have spread north towards Mali and south towards Liberia. The Guekedou area would have emitted several variants towards the north (Faranah), north-east (Kissidougou), east (Macenta and Zorzor) and north-east (Kabala). From Kabala, the virus would have dispersed to coastal Guinea at Madina Oula while undergoing host transfer to *M. erythroleucus* (see Figures S1–S2) and latterly to Makeni. Between Guekedou and Kabala, the virus would have spread to the Bo region, and most recently to the Kenema region. Surprisingly, there is no obvious link between the variants in Faranah in southwestern Guinea and Kabala in northern Sierra Leone, even though the two cities are only 130 km apart. The villages that are closest to each other (Falaba in Sierra Leone and Damania in Guinea) are only 50 km apart and yet show very divergent strains, suggesting that LASV is not spreading through the border. Overall, the migration diagram for the S segment indicates that LASV has spread over a distance of 550 km in 350 years, implying a mean speed of 1.6 km/year (Figure 3c). The migration diagram for the L segment indicates that LASV has spread over a distance of 550 km in 500 years, implying a mean speed of 1.1 km/year (Figure 4c).

## Discussion

### Host range expansion of LASV

Our study identified two *Lophuromys sikapusi* animals hosting LASV in southern Sierra Leone. This is not necessarily surprising, given that a recent survey in the Bo area indicated an arenavirus IgG prevalence of 1.1% (2/174, [22]). This species was common in the surrounding cultivated region and in the lowlands, reaching 12% (174/1473) of the small mammal population in the Bo area [22]. Also, LASV IgG-positive *L. sikapusi* animals have been observed at a level of 3.4% (2/58) in Côte d’Ivoire [41], implying the species competency to host the virus. Our discovery introduces *L. sikapusi* as a new host reservoir for LASV and aligns with expanding knowledge on the host range of this important virus in West Africa.

Our study also found a *Mastomys erythroleucus* hosting LASV in central Sierra Leone, thus confirming previous studies identifying this species as a host reservoir in Guinea and Nigeria [19]. These studies showed that *M. erythroleucus* served as a reservoir for LASV lineages II, III and IV. In localities where *M. erythroleucus* was infected with LASV, prevalence varied between 5 and 37% (3/63 in Onmba-abena or 10/68 in Ebudin in Nigeria, and 6/16 in Madina Oula in Guinea), indicating that this species is highly competent for the virus. According to phylogenetic studies, it seems that this species may have only recently acquired the capacity to act as a reservoir, for approximately 50 years in Madina Oula in Guinea, 10 years in Onmba-abena and 10 years in Ebudin in Nigeria [42,43]. Furthermore, the level of antibodies against LASV was significant, varying from 7 to 18% (4/34 in Upper Guinea, 2/14 in Bo, Sierra Leone and 11/63 in Onmba-abena, Nigeria) suggesting active circulation of the virus in that species [22,44,45]

To date, five rodent species have been identified as potential reservoirs for LASV: two commensal species (*M. natalensis* and *M. erythroleucus*) and three other species (*H. pamfi*, *Mus baoulei* and *L. sikapusi*) [20,46]. As the latter species are restricted to crops, tall grasses or forests and do not enter dwellings, the risk of direct transmission to humans in the domestic environment is likely to be low. However, this species is highly prized as bushmeat, because of its plump appearance and relatively large size for a mouse. Therefore, if *L. sikapusi* is hunted and consumed as a source of protein, the risk of transmission to humans may be significant. Indirect transmission to commensal rodents is also possible, as they frequent the same habitats at certain times of the year, particularly during the rainy season when food is abundant in gardens and surrounding crops [47].

The LASV clades carried by *H. pamfi* and *M. baoulei* are so different from those carried by *M. natalensis* and *M. erythroleucus* that they cannot be considered direct sources of the strains observed in humans. In contrast, the LASV strains found in *L. sikapusi* in our study clearly belong to the Bo clade, suggesting a host transfer to or from *M. natalensis* or to or from *Homo sapiens*. However, there is insufficient information to imply the direction of transfer. In summary, we conclude that three rodent species (*M. natalensis*, *M. erythroleucus* and *L. sikapusi*) carry phylogenetically very similar LASV strains. This constitutes an increased pool of host reservoirs and raises the possibility of an expansion of human infections due to multiple rodent sources.

### Spread of LASV in the MRU area

As our analysis includes many rodent-derived LASV sequences, we can assume that the geographical clusters are more robust than those observed when only human-derived sequences are used. Indeed, the geographical origin of rodents cannot be doubted, whereas that of humans is sometimes uncertain, due in particular to their frequent movements. Our new rodent-derived sequences reveal a wide diversity of strains circulating in Faranah, Kabala, Gueckedou, Bo and Kenema. In our model, the Faranah clade for example, consists of LASV strains that have evolved solely in Upper Guinea for 100 years. Curiously, this zone is not transmitting variants to other zones. This could be due to a sampling bias, as no rodent sampling was done in a proximal zone such as Kissidougou. In contrast, the Kabala cluster in northern Sierra Leone, which emerged 150 years ago, appears to be emitting numerous variants to the north, south and west.

The Faranah region in upper Guinea and the Kabala area in northern Sierra Leone share a common border. Human migration between the two regions is frequent, notably for trading and family reunions. As a result, it was hypothesized that the LASV strains in the two nations had a continuing genetic link via the highly endemic Faranah region. Surprisingly, the sequences from both locations are phylogenetically distant. In fact, the Kabala clade, is phylogenetically near to the Macenta, Guekedou and Kissidougou clades, suggesting that the virus spread first into northern Sierra Leone from the Forest Guinea region.

Furthermore, the Kabala clade clusters with *M. erythroleucus*-derived sequences from Madina Oula in coastal Guinea. In the instance of the Madina Oula sequences, a human-assisted spread of the virus in this area is assumed due to the absence of *M. natalensis* and the village being situated on the border with Sierra Leone and serving as a stopover for travellers. This is supported further by the clustering of the Madina Oula strains with the Makump strain, located in northern Sierra Leone near a crucial trade route connecting coastal Guinea. Initially, this clade was referred to as the Coastal Guinea clade. We suggest renaming it the Kabala clade, based on the phylogenetic clustering of sequences described in our study.

In our modelling, the Bo clade descended from the same ancestral strain as the Kabala clade about 175 years ago, implying that both clades evolved concomitantly. Some novel rodent-derived strains from Barlie, Yakaji and Nyandeyama strongly clustered with human-derived strains from the Kenema laboratory, suggesting that the patients were infected in the Bo area and treated at the Kenema hospital. This is consistent with an earlier study on the circulation of the virus in the Bo area [22].

The Kenema clade appeared 80–85 years ago, which is very recent in the spread of LASV. Indeed, our findings contradict the commonly held belief that the virus spread from Kenema to other regions of Sierra Leone or by going through the border to Guinea [48]. Thus, we discovered in our study that the strains observed in Denguedou cluster with the Kissidougou clade and do not come from Kenema, Bo or Kabala. Consequently, they are not the result of population movements directly from Sierra Leone during the 1991–2002 civil war. If we observe a recent spread of LASV in Denguedou, of the order of 25–30 years, the reason would rather be linked to population movements within the Parrot's beak. This would appear to be an indirect consequence of the conflict in Sierra Leone, which has caused great instability in the sub-region, particularly in Forest Guinea and the Eastern Province of Sierra Leone. Although belonging to two different chiefdoms, Kenema and Bo are very close (∼60 km) and are inhabited by the Mende and Kono communities. These communities are known for their involvement in diamond mining in the alluvial lowlands, where the first diamonds were found in the 1930s [49]. Since then, mining has continued to expand, employing more and more people. We hypothesize that this vast movement of people (over 75,000) since the 1930s has contributed to the intense spread of the LASV in the Eastern Province of Sierra Leone. The emergence of the Kenema clade could simply reflect human mobility in this region over the last 90 years. There are now several hypotheses as to how the virus spreads: (i) passive transport of infectious rodents; (ii) transport of foodstuffs or surfaces soiled by infectious rodents; and (iii) transport of infectious people. Human-to-human transmission is highly suspected since a recent follow-up study has shown the persistence of the virus in seminal fluid for at least 9 months after the acute phase in survivors [50]. Sexual transmission had already been suggested by McCormick when, in the 1980s, his team noticed more cases coming from people sharing the same room [25]. This is in line with the theoretical assessment of human-to-human transmission reaching 20% of contaminations [51]. In our study, the few rodent-derived sequences from the Kenema area show a very recent insertion into the clade, of the order of 30–40 years (compared with 80 years for human-derived sequences). This also suggests that humans could contaminate their environment, particularly commensal rodents such as *M. natalensis*, and thus initiate a reverse transmission (spillback) from humans to rodents [43].

## Conclusion

The LASV sequences identified in our study contribute novel insights into the spatial variability, emergence and spread of LASV across Guinea and Sierra Leone. Our phylogeographic modelling within the Mano River region suggests that LASV originated in Forest Guinea approximately 320–370 years ago, exhibiting subsequent dispersion in multiple directions, including Phebe, Ganta, Faranah, Macenta and Guekedou. This comprehensive diffusion from a common ancestor in Forest Guinea presents a more nuanced evolutionary scenario for LASV compared to the previously proposed notion of its primordial entry via Liberia [23]. Furthermore, the discovery of *L. sikapusi* and *M. erythroleucus* as new hosts for LASV in Sierra Leone, alongside the well-established *M. natalensis*, unveils a heightened level of virus sharing within the rodent community expanding our understanding beyond previous knowledge. Considering the estimated spread rate of 1.1 to 1.6 km per year and the inclusion of these new reservoirs, we anticipate a denser distribution of the virus, connecting currently dispersed hotspots. Employing predictive modelling can help gauge the extent of this phenomenon, allowing for proactive measures and preparedness against potential epidemics in the region.

## Supplementary Material

Supplementary_figures

Table_S2_S5_sequences_in_this_study

TEMI_236052257_Supplementary_information_R1
