# Supplementary figures and images for: Spatio-temporal spread of Lassa virus and a new rodent host in the Mano River Union area, West Africa
Source: Emerg Microbes Infect. 2023 Dec 4;13(1):2290834. doi: 10.1080/22221751.2023.2290834 (PMC10919312; doi:10.1080/22221751.2023.2290834)

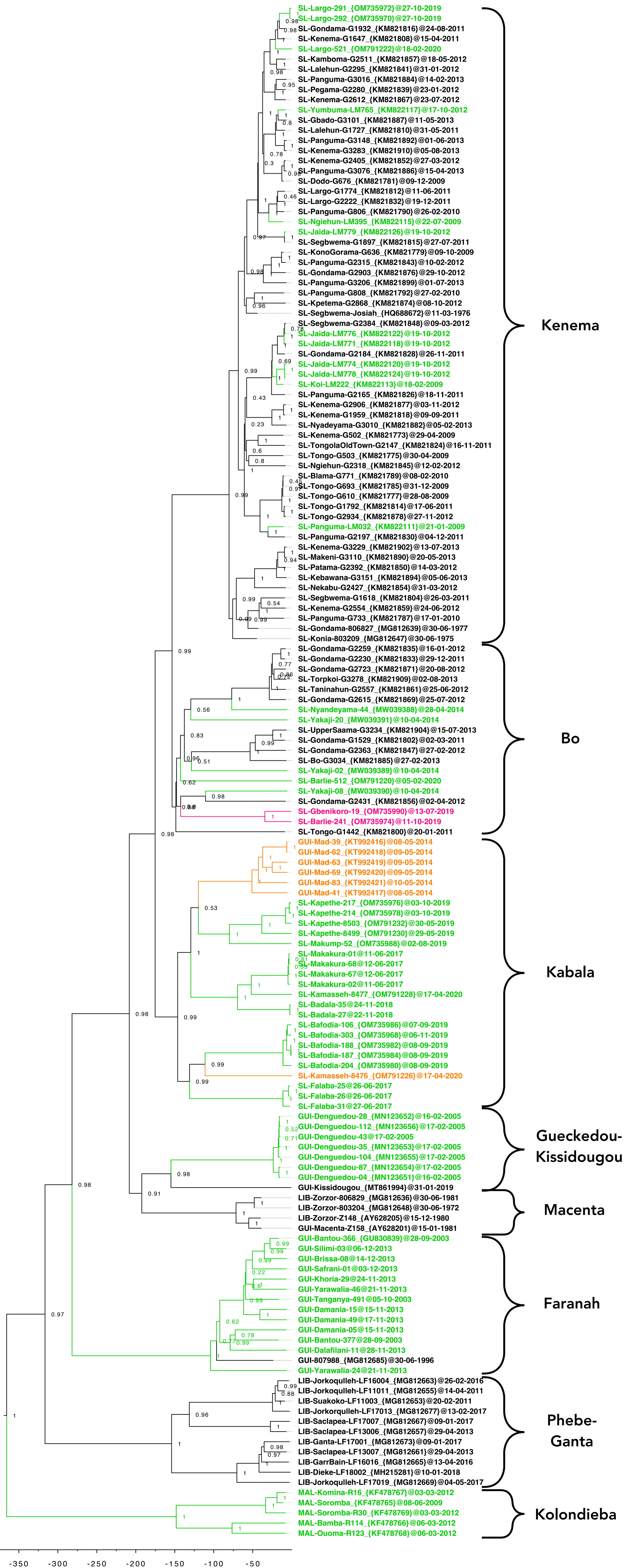

Supplement: Supplementary_figures [file TEMI_A_2290834_SM6031.zip › Fig S1_tree model 3 GP+NP colored.pdf]

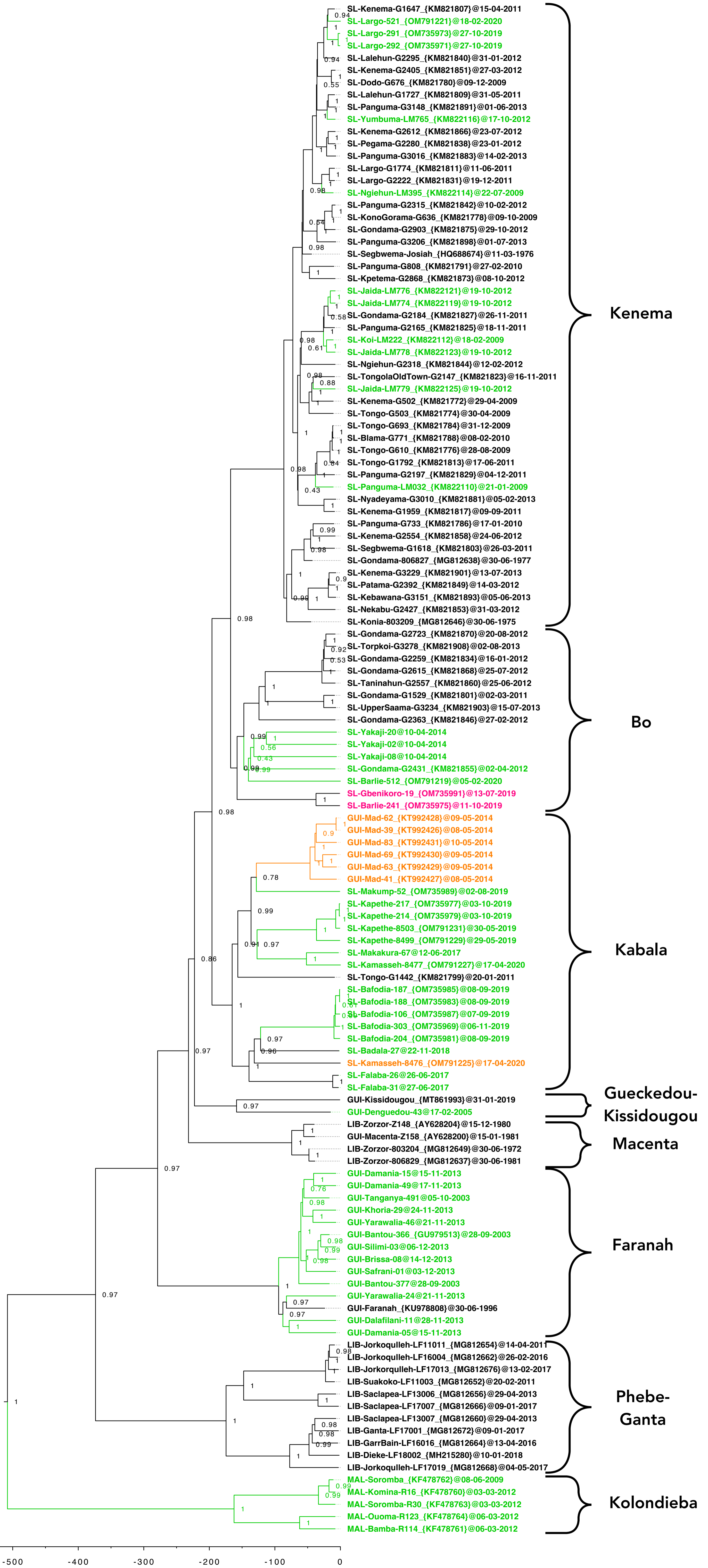

Supplement: Supplementary_figures [file TEMI_A_2290834_SM6031.zip › Fig S2_tree model 6 polymerase colored.pdf]
