# Supplementary material for: Spatio-temporal spread of Lassa virus and a new rodent host in the Mano River Union area, West Africa
Source: Emerg Microbes Infect. 2023 Dec 4;13(1):2290834. doi: 10.1080/22221751.2023.2290834 (PMC10919312; doi:10.1080/22221751.2023.2290834)
Supplement: TEMI_236052257_Supplementary_information_R1 [file TEMI_A_2290834_SM6012.docx]

**Supplementary information**

**Spatio-temporal spreading of Lassa virus and new rodent host in the Mano River Union area, West Africa**

Umaru Bangura^1^, Christopher Davis^2^, Joyce Lamin^3^, James Bangura^4^, Barré Soropogui^5^, Andrew Davison^2^, Jenna Nichols^2^, Matej Vucak^2^, Mickael Dawson^3^, Rashid Ansumana^3^, Dianah Sondufu^3^, Dániel Cadar^1^_,_ Toni Rieger^1^, Emma Thomson^2^, Foday Sahr^6^, N’Faly Magassouba^5^, Bruno Ghersi^7^, Brian H. Bird^7^ and Elisabeth Fichet-Calvet^1^

Table S1. Oligosequence primers and PCR assays used in this study.

| **Primer Pairs** | **Oligosequence, 5’→3’** | **Target gene** | **Reference** |
| --- | --- | --- | --- |
| L7 | ACC AAT GAC ATG AAA AAT CAT CGT T | **Cytochrome b** | ([Ducroz, Granjon et al. 1997](#_ENREF_2)) |
| H15915 | TCT CCA TTT CTG GTT TAC AAG AC |  | ([Ducroz, Granjon et al. 1997](#_ENREF_2)) |
| F-49 | CAT TCA TTG ACC TAC CTG CT |  | ([Lecompte, Brouat et al. 2005](#_ENREF_5)) |
| R-505 | AGA ATC CCC CTC AAA TTC AC |  | ([Lecompte, Brouat et al. 2005](#_ENREF_5)) |
| F-607 | 5'-CGG GCT CTA ATA ACC CAA CG |  | ([Lecompte, Brouat et al. 2005](#_ENREF_5)) |
| R-813 | TTC TGG TTT GAT ATG GGG AGG T |  | ([Lecompte, Brouat et al. 2005](#_ENREF_5)) |
| LVS 36+ | ACC GGG GAT CCT AGG CAT TT | **GPC** | ([Olschlager, Lelke et al. 2010](#_ENREF_7)) |
| LVS-339-d | GTT CTT TGT GCA GGA (AC)AG GGG CAT (GT)GT CAT |  | ([Olschlager, Lelke et al. 2010](#_ENREF_7)) |
| LVS 612+ | ACCTTCATGAGRATGGCTTGGGG |  | ([Olayemi, Cadar et al. 2016](#_ENREF_6)) |
| LVS 732+ | ﻿CCARAACACCACCTGGGAAGATCAYTG |  | ([Olayemi, Cadar et al. 2016](#_ENREF_6)) |
| OWS 1000- | AGCATGTCACAGAAYTCYTCATCATG |  | ([Ehichioya, Hass et al. 2011](#_ENREF_3)) |
| OWS 1430- | CCTWYTATGTGYCTATGTGTTGG |  | ([Ehichioya, Hass et al. 2011](#_ENREF_3)) |
| LVS 1474- | atgCCCATGTGrTTsAGyCTrTG |  | ([Olayemi, Cadar et al. 2016](#_ENREF_6)) |
| LVS 1607+ | GGTGTTGATGTTCTAAASACC | **NP** | ([Olayemi, Cadar et al. 2016](#_ENREF_6)) |
| LVSsl 1607+ | ﻿GGTGTYGATGTTCTRAACACC |  | This study |
| LVS 1629+ | TGTCTCTGGGCAGCACTGCTC |  | ([Fichet-Calvet, Olschlager et al. 2016](#_ENREF_4)) |
| LVS 1673+ | ﻿CCCGACACTGCTGCATCAAACATG |  | This study |
| LVS 2006+ | ﻿CCATGWGAATAYTTGGCATCYTG |  | This study |
| LVSsl 2257RY+ | ﻿CTYCCARCTTTCTGTGGYTT |  | This study |
| OWS 2165A+ | TCT TCA GGT CTC CCT TCW ATG TCN ATC CAN GT |  | ([Ehichioya, Hass et al. 2011](#_ENREF_3)) |
| OWS 2165B+ | TCT TCA GGT CTC CCT TCW ATG TCN ATC CA |  | ([Ehichioya, Hass et al. 2011](#_ENREF_3)) |
| OWS 2170+ | CTC CCT TCW ATG TCN ATC CAN GT |  | ([Ehichioya, Hass et al. 2011](#_ENREF_3)) |
| LVS 2535- | GCCTGCATGTTGGATGGTGGC |  | ([Fichet-Calvet, Olschlager et al. 2016](#_ENREF_4)) |
| LVSsl 2535- | GCYTGCATGYTGGATGGTGGTAAC |  | ([Bangura, Buanie et al. 2021](#_ENREF_1)) |
| OWS 2805+ | GTC AGG CTT GGC ATT GTC CCA AAC TGR TTR TT |  | ([Ehichioya, Hass et al. 2011](#_ENREF_3)) |
| OWS 2810+ | CTT GGC ATT GTC CCA AAC TGR TTR TT |  | ([Ehichioya, Hass et al. 2011](#_ENREF_3)) |
| OWS 2840A- | AAY AAY CAG TTT GGG ACN ATG CCA AG |  | ([Ehichioya, Hass et al. 2011](#_ENREF_3)) |
| OWS2840B- | AAY AAY CAG TTT GGG ACN ATG CC |  | ([Ehichioya, Hass et al. 2011](#_ENREF_3)) |
| LVSsl 3051- | ﻿ACAGAAGAGTGTGCTGAGAG |  | This study |
| OWS 3400- | GCG CAC AGT GGA TCC TAG GC |  | ([Ehichioya, Hass et al. 2011](#_ENREF_3)) |
| OWS 3400A- | CGC AGA GTG GAT CCT AGG CTA TTK GAT TGC GC |  | ([Ehichioya, Hass et al. 2011](#_ENREF_3)) |
| LVL 0003+ | ﻿CGCACCGAGGATCCTAGGCATT | **L** | ([Olayemi, Cadar et al. 2016](#_ENREF_6)) |
| LVL 602+ | ﻿AATGAACAGATACCTGAYTGGG |  | ([Olayemi, Cadar et al. 2016](#_ENREF_6)) |
| LVL 715- | ﻿GAGTCTGCTATACTCTGACTCTTCAA |  | ([Olayemi, Cadar et al. 2016](#_ENREF_6)) |
| LVL 807a- | ﻿CTTGTTGTTAATGCCYTGRTGAC |  | This study |
| LVL 1118+ | ﻿CAAATGCARTGYTGGAGATC |  | ([Olayemi, Cadar et al. 2016](#_ENREF_6)) |
| LVL 1334- | ﻿TCTTTAGATCYCTTTTTGTTGANTC |  | ([Olayemi, Cadar et al. 2016](#_ENREF_6)) |
| OWL 1800+ | ﻿TGTTCATTYTATGCAGATCCTAAAAG |  | ([Ehichioya, Hass et al. 2011](#_ENREF_3)) |
| LVL 1886- | ﻿CATAAAATGAGCARACYTCWCCAAC |  | This study |
| LVL 2320a+ | ﻿GATCAGATAAAATGTTTTGARAAG |  | ([Olayemi, Cadar et al. 2016](#_ENREF_6)) |
| LVL 2665c- | ﻿TCCTTTTCCTTAAATAGWGARCCATTGTT |  | ([Olayemi, Cadar et al. 2016](#_ENREF_6)) |
| LVL 2774a+ | ﻿TATGAATATAAAGTGCAACARGCRATGAG |  | ([Olayemi, Cadar et al. 2016](#_ENREF_6)) |
| LVL 3078- | ﻿TCAAAATCTTCAACCATRTGRT |  | ([Olayemi, Cadar et al. 2016](#_ENREF_6)) |
| LVL 3569a+ | ﻿GGTGACCTTAGGACAAARATGTTTAC |  | ([Olayemi, Cadar et al. 2016](#_ENREF_6)) |
| LVL 3359D_Y+ | AGAATCAGTGAAAGGGAAAGCAAYTC |  | ([Vieth, Drosten et al. 2007](#_ENREF_8)) |
| LVL_3359G Y+ | AGAATTAGTGAAAGGGAGAGTAAYTC |  | ([Vieth, Drosten et al. 2007](#_ENREF_8)) |
| LVL 3569a+ | ﻿GGTGACCTTAGGACAAARATGTTTAC |  | This study |
| LVL 3750- | ﻿GGGCCCCATTTACTRTGRTCCAT |  | ([Olayemi, Cadar et al. 2016](#_ENREF_6)) |
| LVL 3754A_R- | CACATCATTGGTCCCCATTTACTATGRTC |  | ([Vieth, Drosten et al. 2007](#_ENREF_8)) |
| LVL 3754D_R- | CACATCATTGGTCCCCATTTACTGTGRTC |  | ([Vieth, Drosten et al. 2007](#_ENREF_8)) |
| LVL 4253+ | ﻿GATCAGCTCAATAARTTTGTGAGYCC |  | ([Olayemi, Cadar et al. 2016](#_ENREF_6)) |
| LVL 4413- | ﻿GCCAGTTGATGTGGCTCTTTRCAYTT |  | This study |
| LVL 4440- | ﻿TGATCAATGATTGTATCAATTGTYTC |  | ([Olayemi, Cadar et al. 2016](#_ENREF_6)) |
| LVL 4847a+ | ﻿CAGAARATTATATACTCAGGTGC |  | ([Olayemi, Cadar et al. 2016](#_ENREF_6)) |
| LVL 4847b+ | ﻿CAAAARATAATYTATTCAGGTGCTGT |  | ([Olayemi, Cadar et al. 2016](#_ENREF_6)) |
| LVL 4992a- | ﻿TGGAATGCAGATTTGTTGATRGCTTC |  | This study |
| OWL 5510- | ﻿AACTTAATYCTRGGTGTCCACYTCAT |  | ([Ehichioya, Hass et al. 2011](#_ENREF_3)) |
| LVL 5520- | ﻿AACGCTACACAGAGATCTAGAAATTT |  | This study |
| LVL 5753b+ | ﻿GGGTCATTCACATGGTTCCCTCA |  | This study |
| LVL 5910- | ﻿TCTATCCATGCATARCCCATCAT |  | ([Olayemi, Cadar et al. 2016](#_ENREF_6)) |
| LVL 6783a- | ﻿AACTCCTCACAYGTYCTGCCYTT |  | ([Olayemi, Cadar et al. 2016](#_ENREF_6)) |

**References for Tables S1-S5**

Andersen, K. G., B. J. Shapiro, C. B. Matranga, R. Sealfon, A. E. Lin, L. M. Moses, O. A. Folarin, A. Goba, I. Odia, P. E. Ehiane, M. Momoh, E. M. England, S. Winnicki, L. M. Branco, S. K. Gire, E. Phelan, R. Tariyal, R. Tewhey, O. Omoniwa, M. Fullah, R. Fonnie, M. Fonnie, L. Kanneh, S. Jalloh, M. Gbakie, S. Saffa, K. Karbo, A. D. Gladden, J. Qu, M. Stremlau, M. Nekoui, H. K. Finucane, S. Tabrizi, J. J. Vitti, B. Birren, M. Fitzgerald, C. McCowan, A. Ireland, A. M. Berlin, J. Bochicchio, B. Tazon-Vega, N. J. Lennon, E. M. Ryan, Z. Bjornson, D. A. Milner, Jr., A. K. Lukens, N. Broodie, M. Rowland, M. Heinrich, M. Akdag, J. S. Schieffelin, D. Levy, H. Akpan, D. G. Bausch, K. Rubins, J. B. McCormick, E. S. Lander, S. Gunther, L. Hensley, S. Okogbenin, C. Viral Hemorrhagic Fever, S. F. Schaffner, P. O. Okokhere, S. H. Khan, D. S. Grant, G. O. Akpede, D. A. Asogun, A. Gnirke, J. Z. Levin, C. T. Happi, R. F. Garry and P. C. Sabeti (2015). "Clinical Sequencing Uncovers Origins and Evolution of Lassa Virus." Cell 162(4): 738-750.

Bangura, U., J. Buanie, J. Lamin, C. Davis, G. N. Bongo, M. Dawson, R. Ansumana, D. Sondufu, E. C. Thomson, F. Sahr and E. Fichet-Calvet (2021). "Lassa Virus Circulation in Small Mammal Populations in Bo District, Sierra Leone." Biology (Basel) 10(1).

Ducroz, J. F., L. Granjon, P. Chevret, J. M. Duplantier, M. Lombard and V. Volobouev (1997). "Characterization of two distinct species of Arvicanthis (Rodentia: Muridae) in West Africa: cytogenetic, molecular and reproductive evidence." Journal of Zoology, London 241: 709-723.

Ehichioya, D. U., M. Hass, B. Becker-Ziaja, J. Ehimuan, D. A. Asogun, E. Fichet-Calvet, K. Kleinsteuber, M. Lelke, J. ter Meulen, G. O. Akpede, S. A. Omilabu, S. Gunther and S. Olschlager (2011). "Current molecular epidemiology of Lassa virus in Nigeria." Journal of Clinical Microbiology 49(3): 1157-1161.

Fichet-Calvet, E., S. Ölschläger, T. Strecker, L. Koivogui, B. Becker-Ziaja, A. Camara, B. Soropogui, N. Magassouba and S. Günther (2016). "Spatial and temporal evolution of Lassa virus in the natural host population in Upper Guinea." Scientific Reports.

Jahrling, P. B., J. Geisbert and M. S. Ibrahim. (2004). "Direct Submission Lassa mammarenavirus." from https://www.ncbi.nlm.nih.gov/nuccore/AY628201.

Lecompte, E., C. Brouat, J. M. Duplantier, M. Galan, L. Granjon, A. Loiseau, K. Mouline and J. F. Cosson (2005). "Molecular identification of four cryptic species of Mastomys (Rodentia, Murinae)." Biochemical Systematics and Ecology 33: 681-689.

Lecompte, E., E. Fichet-Calvet, S. Daffis, K. Koulemou, O. Sylla, F. Kourouma, A. Dore, B. Soropogui, V. Aniskin, B. Allali, S. Kouassi Kan, A. Lalis, L. Koivogui, S. Gunther, C. Denys and J. ter Meulen (2006). "Mastomys natalensis and Lassa fever, West Africa." Emerging Infectious Diseases 12(12): 1971-1974.

Magassouba, N., E. Koivogui, S. Conde, M. Kono, M. Koropogui, B. Soropogui, I. Kekoura, J. Hinzmann, S. Günther, S. Keita, S. Duraffour and E. Fichet-Calvet (2020). "A sporadic and lethal Lassa fever case in Forest Guinea, 2019." Viruses 12.

Olayemi, A., A. S. Adesina, T. Strecker, N. Magassouba and E. Fichet-Calvet (2020). "Determining Ancestry between Rodent- and Human-Derived Virus Sequences in Endemic Foci: Towards a More Integral Molecular Epidemiology of Lassa Fever within West Africa." Biology (Basel) 9(2).

Olayemi, A., D. Cadar, N. Magassouba, A. Obadare, F. Kourouma, A. Oyeyiola, S. Fasogbon, J. Igbokwe, T. Rieger, S. Bockholt, H. Jerome, J. Schmidt-Chanasit, M. Garigliany, S. Lorenzen, F. Igbahenah, J. N. Fichet, D. Ortsega, S. Omilabu, S. Gunther and E. Fichet-Calvet (2016). "New Hosts of The Lassa Virus." Sci Rep 6: 25280.

Olschlager, S., M. Lelke, P. Emmerich, M. Panning, C. Drosten, M. Hass, D. Asogun, D. Ehichioya, S. Omilabu and S. Gunther (2010). "Improved detection of Lassa virus by reverse transcription-PCR targeting the 5' region of S RNA." J Clin Microbiol 48(6): 2009-2013.

Safronetz, D., N. Sogoba, J. E. Lopez, O. Maiga, E. Dahlstrom, M. Zivcec, F. Feldmann, E. Haddock, R. J. Fischer, J. M. Anderson, V. J. Munster, L. Branco, R. Garry, S. F. Porcella, T. G. Schwan and H. Feldmann (2013). "Geographic distribution and genetic characterization of Lassa virus in sub-Saharan Mali." PLoS Neglected Tropical Diseases 7(12): e2582.

Vieth, S., C. Drosten, O. Lenz, M. Vincent, S. Omilabu, M. Hass, B. Becker-Ziaja, J. Ter Meulen, S. T. Nichol, H. Schmitz and S. Gunther (2007). "RT-PCR assay for detection of Lassa virus and related Old World arenaviruses targeting the L gene." Transactions of the Royal Society of Tropical Medicine and Hygiene 101(12): 1253-1264.

Welch, S. R., W. G. Davis, K. Patel, C. G. Albarino and U. Stroher. (2018). "Lassa virus diversity in Liberia."

Whitlock, A. O. B., B. H. Bird, B. Ghersi, A. J. Davison, J. Hughes, J. Nichols, M. Vucak, E. Amara, J. Bangura, E. G. Lavalie, M. C. Kanu, O. T. Kanu, A. Sjodin, C. H. Remien and S. L. Nuismer (2023). "Identifying the genetic basis of viral spillover using Lassa virus as a test case." R Soc Open Sci 10(3): 221503.

Wiley, M. R., L. Fakoli, A. G. Letizia, S. R. Welch, J. T. Ladner, K. Prieto, D. Reyes, N. Espy, J. A. Chitty, C. B. Pratt, N. Di Paola, F. Taweh, D. Williams, J. Saindon, W. G. Davis, K. Patel, M. Holland, D. Negron, U. Stroher, S. T. Nichol, S. Sozhamannan, P. E. Rollin, J. Dogba, T. Nyenswah, F. Bolay, C. G. Albarino, M. Fallah and G. Palacios (2019). "Lassa virus circulating in Liberia: a retrospective genomic characterisation." Lancet Infect Dis 19(12): 1371-1378.
